# A novel pyroptosis gene expression-based risk score for survival in gastric cancer

**DOI:** 10.3389/fendo.2023.1120216

**Published:** 2023-01-30

**Authors:** Jiali Hu, Yang Song, Xintian Cai, Halike Halina, Kun Qiao, Jiajie Lu, Chengliang Yin, Feng Gao

**Affiliations:** ^1^ Department of Gastroenterology, People’s Hospital of Xinjiang Uygur Autonomous Region, Urumqi, China; ^2^ Xinjiang Clinical Research Center For Digestive Disease, Urumqi, China; ^3^ Department of Gastroenterology and Hepatology, Chinese People's Liberation Army (PLA) General Hospital, Beijing, China; ^4^ Department of Graduate School, Xinjiang Medical University, Urumqi, China; ^5^ Faculty of Medicine, Macau University of Science and Technology, Macao, Macao SAR, China

**Keywords:** gastric cancer, prognostic prediction, signature, pyroptosis, biomarker, LncRNA content

## Abstract

**Background:**

Gastric cancer (GC) is a highly heterogeneous disease, which makes treatment and prognosis prediction difficult. Pyroptosis plays a vital role in the development of GC and influence the prognosis of GC. Long non-coding RNAs (lncRNAs), as regulators of gene expressions, are among putative biomarkers and therapeutic targets. However, the importance of pyroptosis-associated lncRNAs is still unclear in predicting prognosis in gastric cancer.

**Methods:**

In this study, the mRNA expression profiles and clinical data of GC patients were obtained from The Cancer Genome Atlas (TCGA) database and the Gene Expression Omnibus (GEO) database. A pyroptosis-related lncRNA signature was constructed based on TCGA databases by using the Least Absolute Shrinkage and Selection Operator (LASSO) method Cox regression model. GC patients from the GSE62254 database cohort were used for validation. Univariate and multivariate Cox analyses were used to determine the independent predictors for OS. Gene set enrichment analyses were performed to explore the potential regulatory pathways. The immune cell infiltration level was analyzed *via* CIBERSORT.

**Results:**

A four-pyroptosis-related lncRNA (ACVR2B-AS1, PRSS30P, ATP2B1-AS1, RMRP) signature was constructed using LASSO Cox regression analysis. GC patients were stratified into high- and low-risk groups, and patients in the high-risk group showed significant worse prognosis in TNM stage, gender, and age. The risk score was an independent predictor for OS by multivariate Cox analysis. Functional analysis indicated that the immune cell infiltrate was different between high- and low-risk groups.

**Conclusion:**

The pyroptosis-related lncRNA prognostic signature can be used for predicting prognosis in GC. Moreover, the novel signature might provide clinical therapeutic intervention for GC patients.

## Introduction

Gastric cancer (GC) is the fourth-largest cause of cancer mortality, with a worldwide incidence of one million new cases and over 700,000 fatalities every year (1, 2). Surgery, chemotherapy, and targeted molecular therapies are now used to treat GC (3). However, the 5-year survival rate of GC patients is less than 30%, lacking identifiable early gastric cancer symptoms (4). Thus, it is critical to find novel prognostic biomarkers of GC patients that may even be used as realistic targets.

Pyroptosis is a sort of programmed cell death that occurs in response to inflammation. Certain inflammasomes could activate it, resulting in the cleavage of gasdermin D and the activation of inactive cytokines such as IL-18 and IL-1 (5). Pyroptosis is a double-edged sword that plays a vital role in carcinogenesis as well as antitumor response at all stages of tumor formation (6). For example, research found that pyroptosis can aid the development of colitis-associated colorectal cancer by releasing HMGB1, which increases tumor cell proliferation through the ERK1/2 pathway (7). During the alternation of the immune microenvironment, pyroptosis presents tumor-promoting effects through activating inflammasome and the release of cytokines (8). Inhibition of GSDMD expression delays pyroptosis and accelerates tumor cell proliferation in gastric cancer through promoting the transition from the S to G2 phase (9). Therefore, pyroptosis is a potential therapeutic target for inhibiting tumor cell growth through promoting pyroptosis. The acute activation of pyroptosis, on the other hand, reduced tumor progression by boosting the immune cell infiltration (10, 11). However, the impact of pyroptosis on the prognosis of GC patients is still unclear.

Long non-coding RNAs (lncRNAs) are a kind of transcript with a total length of more than 200 nucleotides. They influence gene expression by chromatin remodeling as well as transcriptional and posttranscriptional changes (12). Aberrant expression of lncRNAs in various cancers has suggested a role in cancer etiology, and this is no exception in GC (13). For example, one research found that MEG3 inhibited gastric cancer growth and metastasis through the p53 signaling pathway (14). Another study discovered that MALAT1 was employed as a competitive endogenous RNA for miR-23b-3p, which contributed to chemo-induced autophagy and chemoresistance in GC cells (15). It was also shown that the levels of lncRNA GASL1 and PTCSC3 expression are connected to tumor size, TNM stage, and GC distant metastases (16). However, the relevance of pyroptosis-related lncRNAs in gastric cancer formation remains unknown and little research has focused on the link between pyroptosis and GC advancement. Understanding the link between pyroptosis-related lncRNAs and GC development may help to find novel biomarkers that may be used as therapeutic targets.

In this study, we constructed a pyroptosis-related lncRNA prognostic signature as an independent prognostic factor with high accuracy in predicting overall survival (OS). Our results showed that this signature was instrumental in the GC tumorigenesis-related pathway and was highly connected with the tumor microenvironment. We believe that the powerful prognostic signature could give a constructive tip for helping to improve risk stratification of gastric cancer patients and provide a more effective assessment for clinical management.

## Results

### Patient public data

A cohort consisting of a total of 250 gastric cancer patients with available expression data and clinical information were obtained from The Cancer Genome Atlas (TCGA) database as the training set. There were 300 patients from the Gene Expression Omnibus (GEO) database GSE62254 cohort enrolled as the validation set. The clinical characteristics of patients in both training and validation cohorts are summarized in **Table 1**. Among the training cohort, the TNM stage was stage I in 14.1%, stage II in 31.3%, stage III in 33.7%, and stage IV in 4.3% of cases. The detailed flowchart of this study is presented in **Figure S1**.

**Table 1:** Baseline clinical characteristics of gastric cancer patients.

Characteristics	TCGA N = 250	GSE62254 N = 300
Age group		
NA	4 (8.9%)	0 (0%)
Young	79 (26.3%)	106 (35.3%)
Old	167 (55.7%)	194 (64.7%)
Gender		
Male	157 (52.3%)	199 (66.3%)
Female	93 (31.0%)	101 (33.7%)
TNM stage		
NA	3 (1.0%)	1 (2.2%)
I	30 (10.0%)	31 (10.3%)
II	94 (31.3%)	163 (54.3%)
III	101 (33.7%)	78 (26.0%)
IV	13 (4.3%)	27 (9.0%)

### Identification of prognostic pyroptosis-related lncRNAs and construction of a prognostic signature in the training cohort

**Figure 1:**
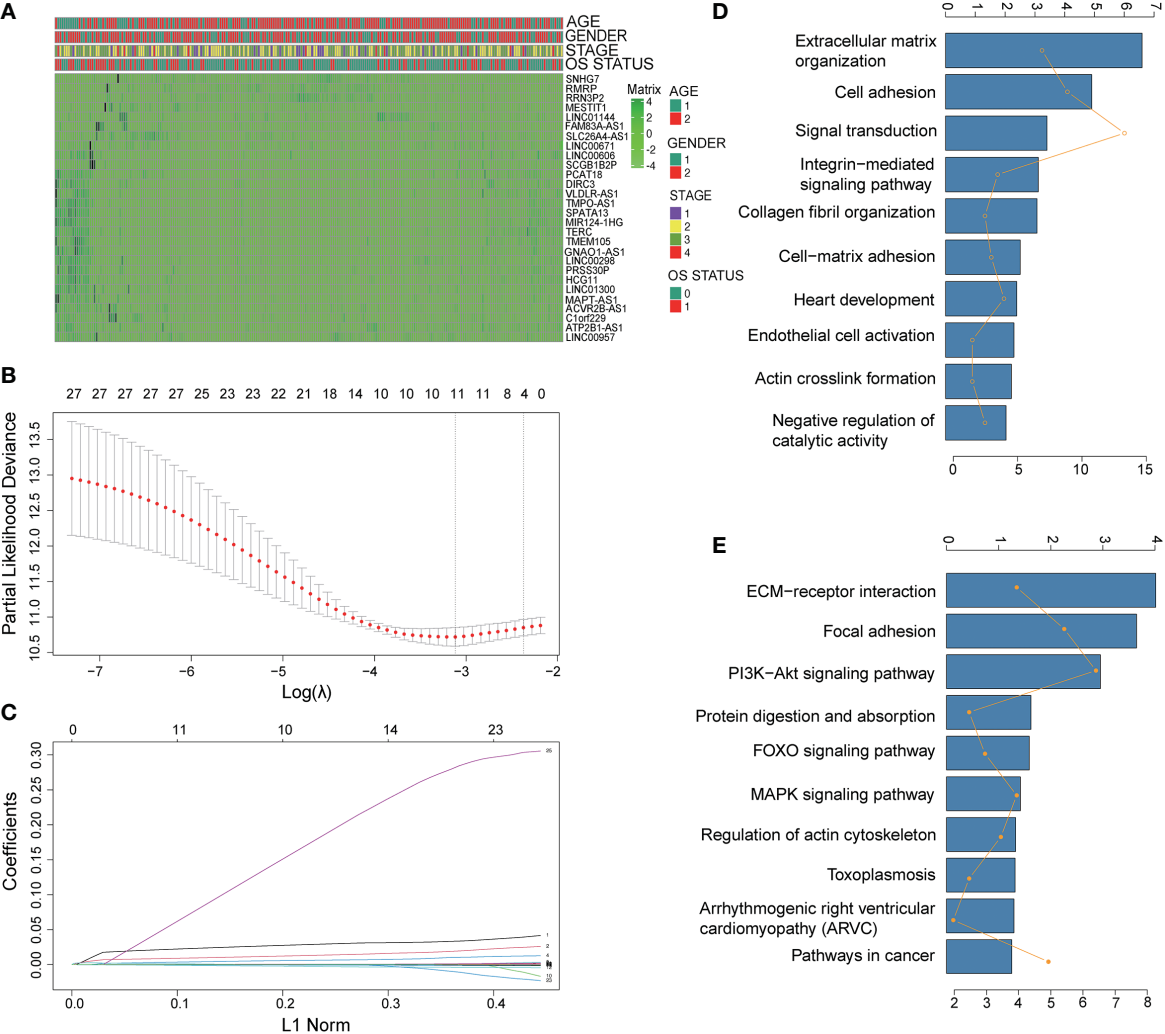
Identification of prognostic pyroptosis-related genes in gastric cancer. **(A)** The heatmap of 28 pyroptosis-related lncRNAs. **(B)** LASSO coefficient profiles of the most useful prognostic genes. **(C)** Cross-validation for tuning parameter selection in the LASSO model. **(D, E)** Gene set enrichment analysis of the lncRNA signature. **(D)**: GO annotation; **(E)** KEGG pathway).

A total of 123 pyroptosis-related lncRNAs were matched with the training gene set (TCGA), 28 of which were correlated with OS in the univariate Cox regression analysis in the training set (**Table S1**). The heatmap of 28 pyroptosis-related lncRNAs is shown in **Figure 1A**. Using deviance as the selection criteria, the LASSO Cox method was used for modeling the process of gene selection. Partial likely deviant distribution of each log (λ) was set out, as shown in **Figure 1B**. The coefficient results in distribution when using different variables are presented in **Figure 1C**. The signature that had the best results included ACVR2B-AS, PRSS30P, ATP2B1-AS1, and RMRP. We further used the four lncRNAs to build a Cox regression model. A four-lncRNA signature of OS was identified based on the optimal value of λ (**Figure 1B**). The risk score was calculated as follows:

Risk score = 0.019344382* ACVR2B-AS1 + 0.000043085 * PRSS30P + 0.006481851* ATP2B1-AS1- 0.001247169 * RMRP

To explore the functional implication of signature, the expression correlation between mRNAs and each of the four lncRNAs in the signature was carried out, and the co-expressed mRNAs were selected. GO term analyses were performed to explore the potential biological functions of the co-expressed mRNAs. As shown in **Figures 1C–E**, terms with more genes tended to have higher p-values, and the GO (http://geneontology.org) (17) annotation and KEGG (http://www.kegg.jp/ or http://www.genome.jp/kegg/) (18) pathway revealed top categories that were positively correlated with, such as extracellular matrix organization, signal transduction, ECM–receptor interaction, and focal adhesion. These results indicated that GO enrichment was critically important in gastric cancer patients.

### Independent prognostic role of the prognostic lncRNA signature

**Figure 2:**
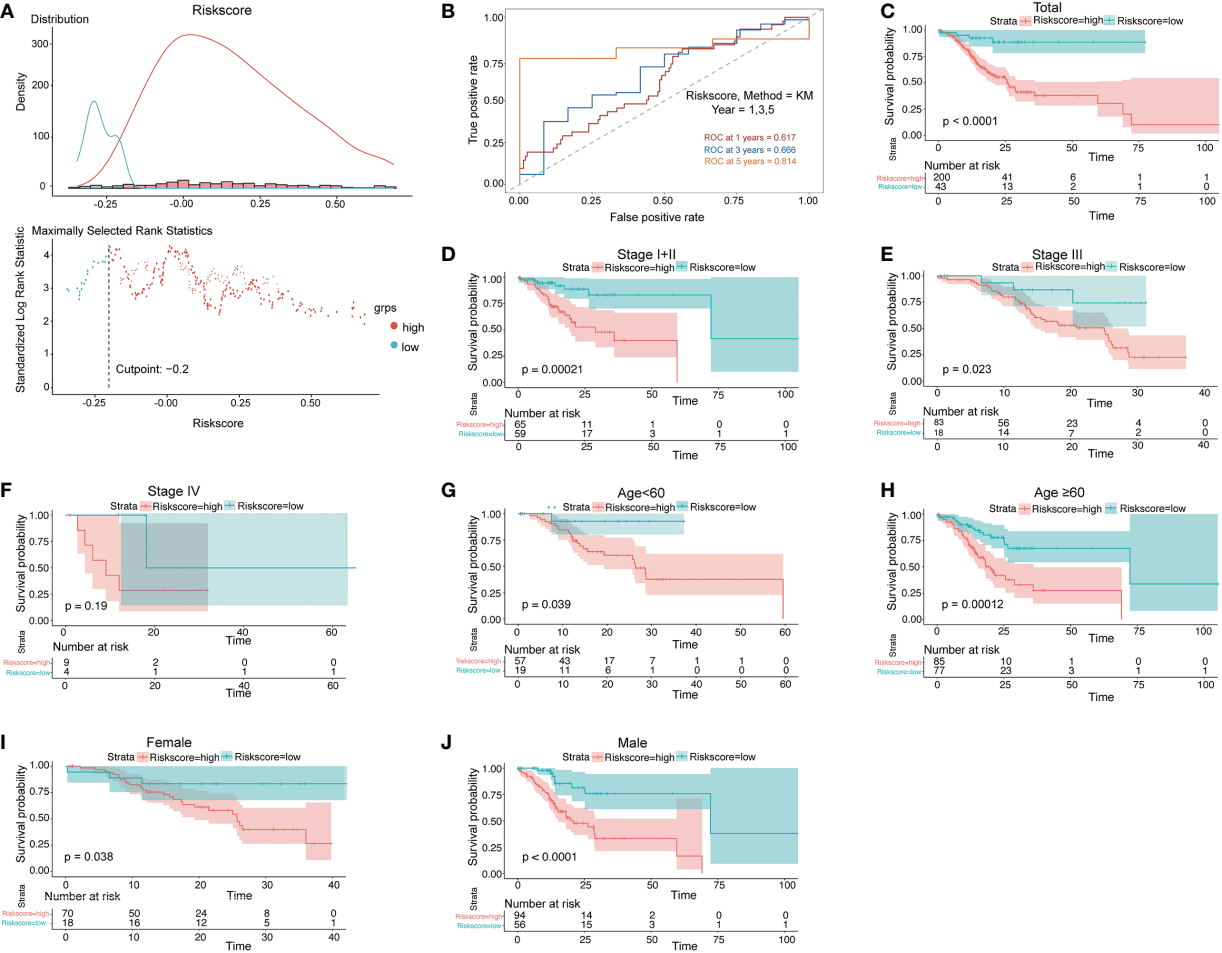
Prognostic OS analysis of the four-gene signature model in the training cohort. **(A)** The distribution and cutoff value of the risk scores in the training cohort. **(B)** AUC of time-dependent ROC curves verified the prognostic performance of the risk score in the training cohort. **(C–F)** Kaplan–Meier curves for the OS of patients in the high-risk group and low-risk group in the TNM stage (total, I+II, III, IV). **(G, H)** Kaplan–Meier curves for the OS of patients in the high-risk group and low-risk group for age (age<60, age ≥60) in the training cohort. **(I, J)** Kaplan–Meier curves for the OS of patients in the high-risk group and low-risk group for gender (female, male) in the training cohort.

**Figure 3:**
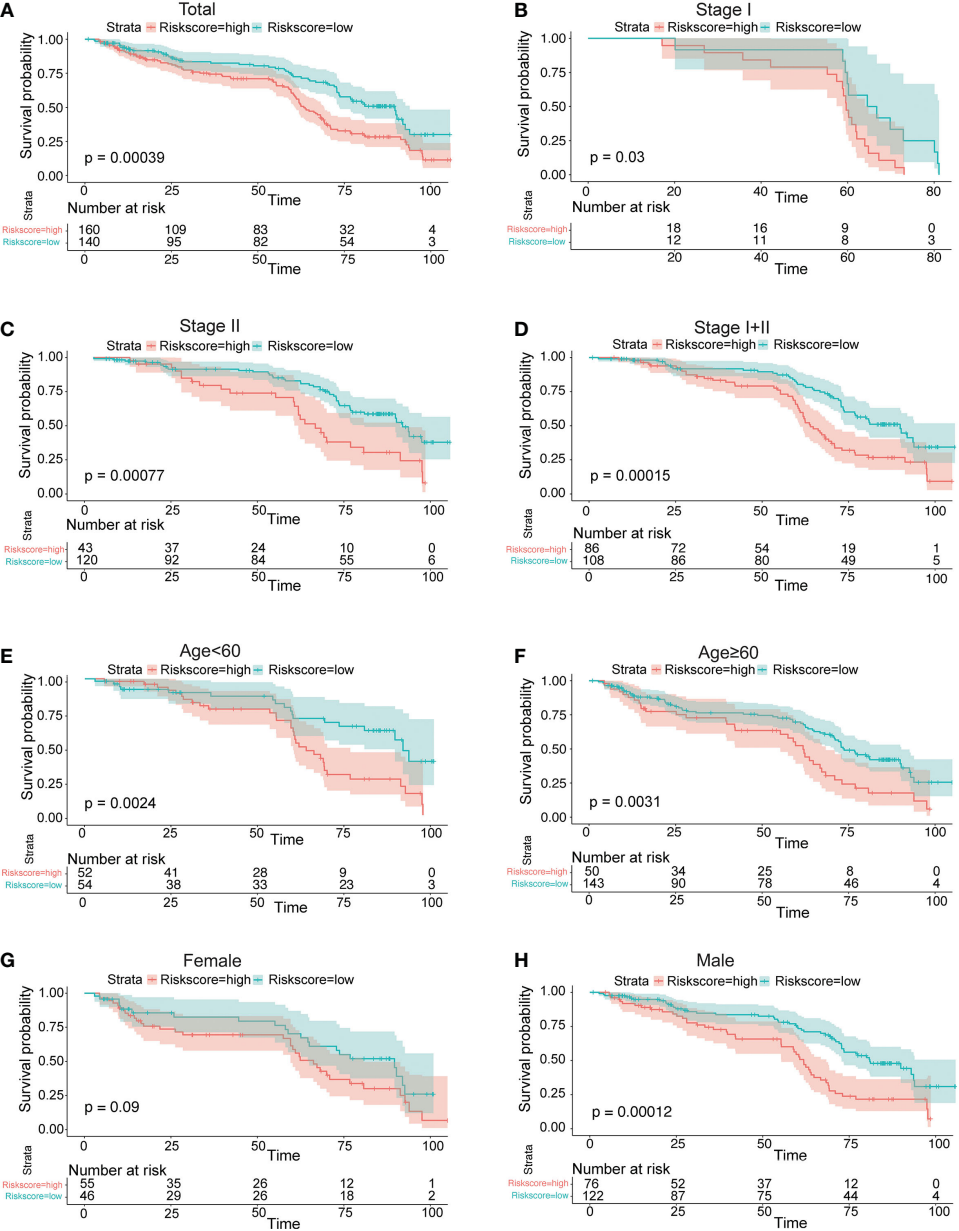
Prognostic OS analysis of the four-gene signature model in the validation cohort in GSE62254. **(A–E)** Kaplan–Meier curves for the OS of patients in the high-risk group and low-risk group in the TNM stage (total, I, II, I+II). **(E, F)** Kaplan–Meier curves for the OS of patients in the high-risk group and low-risk group for age (age<60, age ≥60) in the training cohort. **(G, H)** Kaplan–Meier curves for the OS of patients in the high-risk group and low-risk group for gender (female, male) in the training cohort.

Survival analyses based on the optimal cutoff expression value of each lncRNA indicated the relationship between high expression of four lncRNAs and their prognosis. The result of the best cutoff value that can separate the people into high-risk and low-risk groups was -0.2, according to the risk score distribution plot in **Figure 2A**. The patients were stratified into high-risk group (n = 43) and low-risk group (n = 200) according to the cutoff value (**Figure 2A**). The high-risk group turned out to be significantly different from the low-risk group in clinical features (TNM stage I+II, stage II, stage III, male, female, young patients, older patients, and total population) in the training cohort (**Figure 2**). As shown in **Figures 2C–F** patients with a high risk had a high probability of death than those with a low risk in the TNM stage (all stages, stage I+II, stage III). The predictive performance of the risk score for OS was evaluated by time-dependent ROC curves, and the area under the curve (AUC) was 0.81 at 5 years (**Figure 2B**). The consistent results of clinical features were seen in the validation (GSE62254) (**Figure 3**).

### Independent prognostic factor role of the four-lncRNA signature

**Figure 4:**
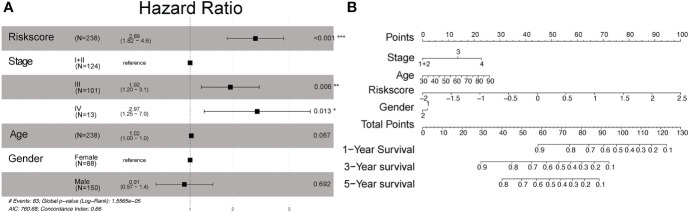
Multivariate Cox proportional hazard models of known clinical risk factors. **(A)** Forest plot of the multivariate Cox regression analysis in gastric cancer training set. **(B)** Establishment of the nomogram predicting OS for gastric cancer patients in the training cohort. The nomogram plot was built based on four prognostic factors in gastric cancer. *P<0.05; **P<0.01; and ***P<0.001.

To ensure the completeness of clinical information, we dropped the patients with a null value in stage, clinically distinct subtypes, age, and risk score, and got 238 patients after this step. The age was separated by 60 as young and old patients. Among the 238 OS patients included in the training dataset, univariate Cox regression analysis indicated that age group, gender, stage, and our prognostic model were significant. Among these factors, TNM stage and risk score were the hazardous variables (p-value<0.05). Also, multivariate Cox regression showed that risk score was an independent prognostic factor for OS of GC (**Figure 4A**).

We then used multivariate analysis to construct a nomogram. It was built by including age, clinically distinct subtypes, stage, and risk score (**Figure 4B**). As shown in **Figure 4B**, the risk signature was the most important factor affecting the patients’ survival, followed by stage and age. In the nomogram, the probability of 1, 3, and 5 years was vertically paralleled with the total points calculated by the sum of points of every single variable.

### External validation using the online database

**Figure 5:**
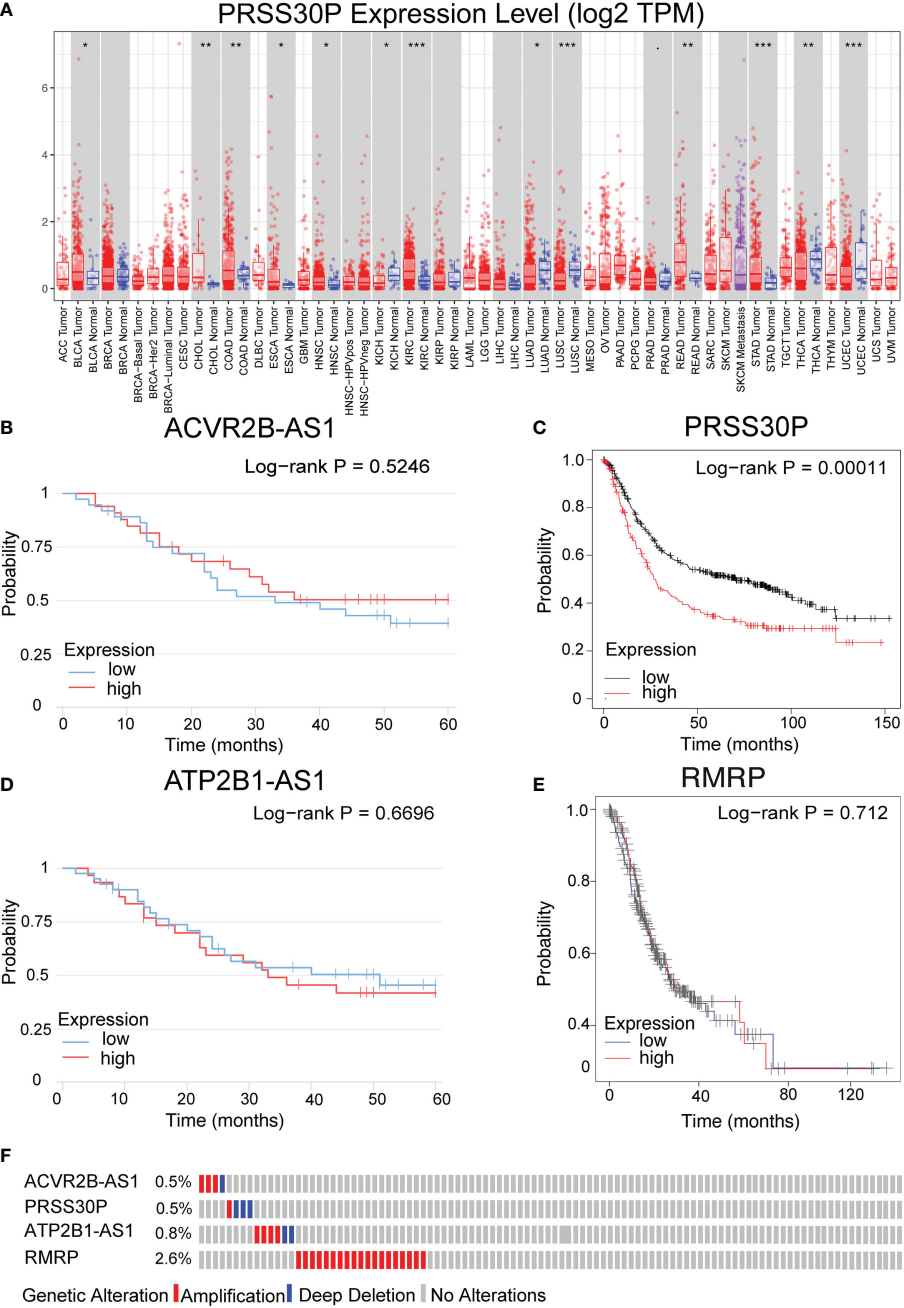
Expression and genetic alterations of the four predictive genes. **(A)** The expression level of PRSS30P in tumor tissue and normal tissue in TIMER (https://cistrome.shinyapps.io/timer/). **(B–E)** Prognostic values of ACVR2B-AS1, PRSS30P, ATP2B1-AS1, and RMRP expression in overall survival. **(F)** Genetic alterations of the four genes. Data were from the cBioPortal for Cancer Genomics (http://www.cbioportal.org/). *P<0.05; **P<0.01; and ***P<0.001.

PRSS30P expression in gastric tumor was higher than normal tissue (**Figure 5A**). The PRSS30P expression patient group displayed remarkable longer OS in the Kaplan–Meier plotter (**Figure 5C**). However, ACVR2B-AS1, ATP2B1-AS1, and RMRP were insignificant differences in OS (**Figure 5B, D, E**). Taken together, PRSS30P was an aberrant expression gene and a low PRSS30P expression predicted adverse outcomes as a potential prognosticator. In cBioPortal (17) for the Cancer Genomics website, RMRP among four genes of the risk score model possessed the most frequent genetic alterations (2.6%), and missense mutation was the most common alteration (**Figure 5F**).

### Immune infiltration using the CIBERSORT database

**Figure 6:**
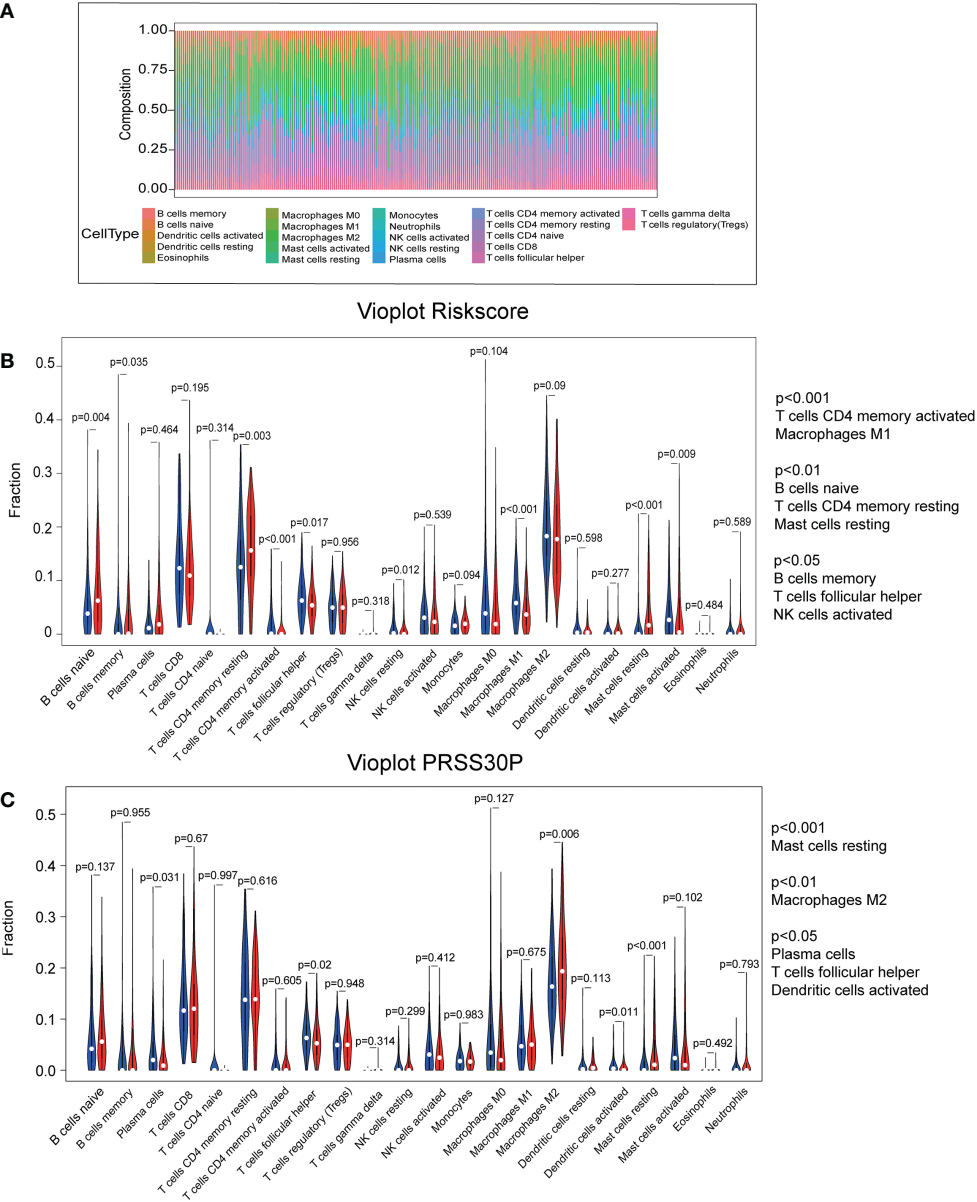
Immune infiltration using the CIBERSORT database. **(A)** The RNA-Seq of TCGA-STAD database was analyzed to obtain the abundance ratio matrix of 22 immune cells *via* CIBERSORT. **(B)** The differential abundance of immune infiltrates was obtained by comparing the distribution of immune cells in low- and high-score groups using R software. **(C)** The differential abundance of immune infiltrates was obtained by comparing the distribution of immune cells in low- and high-PRSS30P groups using R software.

As shown in **Figure 6A**, the abundance ratio of 22 immune cells in the 250 GC samples was analyzed by CIBESORT (18). It can be seen from the figure that M2 macrophages and resting CD4 memory T cells were the most abundant compared with other immune cells. The results of the correlational analysis of immune cells are presented in **Figure S2**. Neutrophils and activated dendritic cells were significantly correlated, whereas resting CD4 memory T cells were negatively correlated with CD8 T cells. Additionally, using the CIBERSORT algorithm, we focused on the difference between high-risk score tissue and matched low-risk score tissue. Memory activated CD4 T cells, M1 macrophages, memory B cells, follicular helper T cells, and activated NK cells were found in higher numbers in high-risk score GC tissue than in matched low-risk score tissue. However, naïve B cells and resting CD4 memory T cells were all lower (**Figure 6B**). We also analyzed the difference between PRSS30P high-expression tumor tissue and paired PRSS30P low-expression tissue using the CIBERSORT algorithm. We found that there were significant differences in follicular helper T cells and resting mast cells between high- and low-risk groups and high- and low-PRSS30P groups.

## Discussion

Pyroptosis is a type of programmed cell death that happens in pathogen-infected cells, triggering the body’s inflammatory response. Pyroptosis has played a dual role in a wide range of malignancies in recent years (19). On the one hand, normal cells are stimulated by a large number of inflammatory factors released by pyroptosis, leading to their transformation into tumor cells (20). On the other hand, the promotion of tumor cell pyroptosis could be a new therapeutic target (21). However, the role of pyroptosis-related lncRNAs in gastric cancer remains unknown. Given the significantly disparate prognosis outcomes of gastric cancer, it is necessary to develop a strong classifier to stratify patients with variable risks and prognoses, which is crucial to maximizing the advantages of customized therapy and early follow-up.

In this study, we systematically investigated the involvement of pyroptosis-related lncRNAs in gastric cancer to help address this important clinical issue. A novel prognostic signature that involved four pyroptosis-related lncRNAs was constructed and validated in an external cohort. Functional analysis revealed that pyroptosis-related pathways were enriched in gastric cancer. These results provide a new insight into the discussions about patient prognosis and stratification by considering pyroptosis and microenvironmental features.

In our work, 28 pyroptosis-related lncRNAs were found to be correlated with OS in the univariate Cox regression analysis. Our prognostic signature was composed of four pyroptosis-related lncRNAs (ACVR2B-AS1, PRSS30P, ATP2B1-AS1, RMRP). In the TNM stage, patients in the high-risk group had a shorter OS than patients in the low-risk group (p< 0.001 in both the training and validation cohorts). Our prognostic signature was shown to be an independent prognostic factor for OS (HR >1, p< 0.010) in multivariate Cox regression analysis. The signature’s predictive ability was proven by ROC curve analysis. Pyroptosis-related pathways were found to be overrepresented in functional analysis. We also created a nomogram that included age, gender, stage, and the signature. Validation cohorts were used to test the nomogram’s performance. Our nomogram may be able to predict the prognosis of stomach cancer simply and reliably.

The signature lncRNAs revealed in this research have previously been linked to important functions in a variety of malignancies. ACVR2B-AS1 (ACVR2B-antisense RNA1) is a newly discovered long non-coding RNA. It is found on 3p22.2 and is transcribed from ACVR2B’s opposing strand. A higher ACVR2B-AS1 expression in liver cancer patients was shown to be an independent unfavorable prognostic factor for overall survival (OS) in a study (22). PRSS30P (serine protease 30, pseudogene) is located on 16.10. A study showed that PRSS30P was shown to improve efficiency in distinguishing sepsis-induced ARDS from sepsis (23). ATPase plasma membrane Ca2+ transporting 1 antisense RNA 1 (ATP2B1-AS1), also known as long intergenic non-protein-coding RNA 936 (LINC00936), has been shown to be a crucial regulator in chronic renal failure-induced renal interstitial fibrosis and oxidative stress (24). It was discovered that upregulated ATP2B1-AS1 or silenced miR-425-3p prevents gastric cancer cells from escaping the immune system by increasing ZC3H12A levels (25). In cartilage-hair hypoplasia (CHH), an autosomal recessive hereditary condition, the RNA component of mitochondrial RNA processing endoribonuclease (RMRP), an lncRNA, was first found (26). It was found that RMRP accelerated tumorigenesis by serving as a miR-206 sponge and that it may be exploited as a new gastric cancer biomarker (27). The four signature lncRNAs, however, have received little attention in the context of combination pyroptosis and immunity. As a result, the signature lncRNAs discovered in this work might offer a key target for laboratory experimental design to unravel the biochemical mechanisms of gastric cancer.

In this research, patients with a low PRSS30P expression had significantly longer OS in the Kaplan–Meier plot (**Figure 6C**). Meanwhile, PRSS30P was discovered to be comparable with OS results in GSE66254. Additionally, in the external validation data, we found that the expression of PRSS30P was higher in gastric cancer tissue than in normal gastric tissue. Therefore, PRSS30P could be a prognostic biomarker for gastric cancer.

Finally, we looked at the relationships between four lncRNAs in the risk score model and 22 different kinds of invading immune cells and discovered that follicular helper T cells and resting mast cells were strongly linked to gastric cancer. Although little is known about the roles of follicular helper T cells and resting mast cells in tumor immune response in gastric cancer, we explored and discovered some. According to one research, dysregulation of follicular helper T subsets in gastric cancer patients, as seen by increased Th1-follicular helper T cells, led to inflammation and tumor formation (28). Another research discovered that circulating follicular helper T (cTfh) cells and their related factors (IL-21/CXCL13) may have a role in the development and progression of gastric cancer (29). Mast cells are tissue-resident, innate immune cells that play an important role in inflammation and tissue homeostasis. Mast cells develop in the stroma of several human cancers, and higher mast cell density has been linked to either a favorable or a bad prognosis, depending on the tumor type and stage (30). According to one study, increased intratumoral mast cells promoted immune suppression and gastric cancer growth through the TNF-PD-L1 pathway (31).

This research has some limitations. Firstly, despite the fact that several independent external validations (TCGA, GEO) were carried out in this investigation, it was impossible to cover all variances among patients from various geographical locations when tissues and information were gathered from publicly accessible databases. Therefore, this pyroptosis prognostic signature needs to be further verified in prospective, multicenter, real-world studies. Second, our analysis only indicated a preliminary link between related lncRNAs and prognosis of gastric cancer. Experiment research is needed to better investigate the fundamental processes.

## Conclusions

In conclusion, we developed a new pyroptosis-related predictive signature in patients with gastric cancer. Pyroptosis-related signature may play a role in antitumor formation and may act as therapeutic targets for gastric cancer.

## Materials and methods

### Patient publicly available data acquisition and pyroptosis gene sets

The training cohort contained 250 gastric cancer patients which were obtained from The Cancer Genome Atlas (TCGA; https://www.cancer.gov/about-nci/organization/ccg/research/structural-genomics/tcga). External validation was downloaded from the Gene Expression Omnibus (GEO; http://www.ncbi.nlm.nih.gov/geo/) database (GSE62254; n = 300). Only patients with sufficient expression profiles, clinicopathologic data, and survival data were included in the analysis. TCGA database, consisting of a total 250 of gastric cancer patients, was used as the training set. Meanwhile, 300 gastric patients from the GSE62254 cohort as the validation set were completely enrolled. The list included 16,887 lncRNAs from the lncRNA annotation file of Genome Reference Consortium Human Build 38 (GRCh38), which was acquired from the GENCODE website. The list of 33 pyroptosis-related genes was gathered from prior reviews (20, 21, 32, 33), which is provided in **Table S1**.

### Data cleaning

Firstly, we matched the TCGA gastric cancer dataset with the clinical dataset. There were 243 people remaining after we removed the population whose OS months were 0 and the survival status of OS months was null. There were 29 intersection genes obtained after pyroptosis-related genes were crossed with the training population genes profile. A total of 372 intersection lncRNAs were obtained after the dataset containing 16,887 lncRNAs were crossed with the training population gene profile. By correlation analysis, 123 pyroptosis-related lncRNAs were screened out. To screen pyroptosis-related lncRNAs with prognostic values, we performed univariate Cox analysis of overall survival (OS). Log-rank tests were used to adjust the significance of the analysis with a p-value less than 0.05. These selected genes were used as candidates for the Lasso Cox regression model. The 28 lncRNAs were used as candidates for the Lasso Cox regression model.

### Construction and validation of a prognostic pyroptosis-related gene signature

Lasso-penalized Cox regression analysis was utilized to select the best variables set and minimize the risk of overfitting. The “glmnet” R package was used to conduct the Least Absolute Shrinkage and Selection Operator (LASSO) algorithm. The normalized expression matrix of candidate prognostic-related genes was regarded as the independent variable and the overall. The survival and status of gastric cancer patients in the training datasets were regarded as the response variables.

Cross-validation following the 1 standard error criteria was used to determine the penalty parameter (λ) for the model. The partial likely deviant distribution of each parameter (λ) and the coefficient result distribution when using different variables were shown as the principle of how the final variables set were selected. The stepwise method was utilized to optimize the variable combination with the lowest AIC value. The risk score of the patients was calculated according to the sum or product of the normalized expression level of each lncRNA in the final set and its corresponding regression coefficients. The formula was established, as follows:


$$Risk\;Score\;=\;\sum_{i=1}^{N}Expi\;\times \;Coefi$$

After we used the formula to calculate the risk score and the “surviminer” R package to find the best cutoff result, a scatter plot was applied to show the distribution of the follow-up month distribution comparison between the high- and low-risk groups. Meanwhile, the cutoff position which separates the high and low in the risk score result was presented in a scatter plot. For the survival analysis of the established model, the optimal cutoff expression value was determined by the “surv_cutpoint” function of the “survminer” R package. The “survivalROC” R package was utilized to conduct time-dependent ROC curve analyses to evaluate the predictive power of the gene signature. To show the function of the established risk score model, we utilized a similar method to show the survival curve difference in TNM stage (stage II, stage III, TNM stage I+II, TNM stage III+IV), age, and gender.

To show the results of the established risk score model correlated with clinical information, we added some analysis by combining the established risk score model with clinical information. To ensure the completeness of data, we deleted the individuals with a null value in engaged variables. The process of analyzing included three steps. First, after removing the single variable with the log-rank value of Cox regression ≤0.05, we built a multivariate model with all statistically significant variables with the obtained risk score and the nomogram of the model. Second, the ROC curve was used to show the time-dependent calibration situation of every single clinical information-based model and the multivariate model with all statistically significant variables with the obtained risk score. In addition, the results of each univariate Cox regression model and the multivariate Cox regression model were shown in a forest plot. We also fit a nomogram plot with the full model to help clinical doctors to understand and apply the model.

### Estimation of risk score signature, construction, and assessment of the nomogram combining signature and clinical information

The Kaplan–Meier method and log-rank test were utilized to estimate the association between risk score and OS. The proportional hazard assumption was confirmed for each variable before fitting Cox models. Multivariate Cox proportional hazard models were utilized to learn the association between risk score levels and OS in the presence of known clinical risk factors. The forest plot was used to show the p-value, HR, and 95% CI of each variable and multivariate model through the “forestplot” R package. We performed the nomogram and evaluated the performance of the 1-, 3-, and 5-year OS predictions. Then, ROC analysis was used to calculate AUC and check the prediction accuracy for the multivariate model.

### Functional enrichment analysis

The expression correlation between mRNAs and each of the four lncRNAs in the model was performed, and the co-expressed mRNAs were selected. Metascape was utilized to conduct Gene Ontology (GO) and Kyoto Encyclopedia of Genes and Genomes (KEGG) analyses based on the co-expressed mRNAs.

### Immune infiltration analysis

CIBERSORT is a powerful analysis tool that employs 547 gene expression signatures. Using a deconvolution technique, it defines each immune cell subtype and precisely measures unique immune cell compositions. The calculated p-value represents the statistical significance of the deconvolution findings and may be used to filter out samples with less significant accuracy. The original TCGA gene expression data were obtained and uploaded to the CIBERSORT web interface (http://cibersort.stanford.edu). For each sample, the relative proportions of 22 invading immune cells, as well as the CIBERSORT metrics of CIBERSORT p-value, Pearson correlation coefficient, and root mean squared error (RMSE), were analyzed concurrently.

### External validation using the online database

Hub lncRNAs in LASSO Cox were surveyed from several online databases as follows: (1) lncRNA expression analysis in TIMER (34). The online database Gene Expression Profiling Interactive. (2) Kaplan–Meier Plotter Database Analysis. The correlation between lncRNA expression and survival in variable cancer was analyzed by the Kaplan–Meier Plotter (http://kmplot.com/analysis/). Kaplan–Meier Plotter searches for relationships between expression and patient prognoses, such as overall survival (OS), across a large collection of publicly available cancer microarray datasets. The threshold was adjusted to a Cox p-value< 0.05. (3) InCAR (https://lncar.renlab.org/) is a comprehensive dataset devoted to displaying differential expression profiles and the prognostic landscape in human malignancies by the reannotation of microarray probes. To investigate the function of relevant lncRNAs, differential expression analysis, survival analysis, co-expression analysis, KEGG pathway enrichment analysis, ceRNA analysis, and meta-analysis were provided by InCAR. (4) cBioPortal analysis. The cBioPortal for Cancer Genomics (http://cbioportal.org) was specifically designed to lower the barriers of access to the complex data sets and thereby accelerate the translation of genomic data into new biological insights, therapies, and clinical trials (35). The portal facilitates the exploration of multidimensional cancer genomics data by allowing visualization and analysis across genes, samples, and data types. Users can visualize patterns of gene alterations across samples in a cancer study, compare gene alteration frequencies across multiple cancer studies, or summarize all relevant genomic alterations in an individual tumor sample. Genomic data types integrated by cBioPortal include somatic mutations, DNA copy-number alteration, mRNA and microRNA expression, DNA methylation, protein abundance, and phosphoprotein abundance.

### Statistical analysis

The Student’s t-test was used to compare gene expression between tumor tissues and adjacent non-tumorous tissues. The OS between different groups was compared by Kaplan–Meier analysis with the log-rank test. Univariate and multivariate Cox regression analyses were implemented to identify independent predictors of OS. All statistical analyses were performed with R software (Version 3.5.3). A p-value less than 0.05 was considered statistically significant.

## Data availability statement

Original data from RNA-Seq are from public data and available in the Gene Expression Omnibus(GEO; https://www.ncbi.nlm.nih.gov/geo/ under the accession number GSE62254), The datasets generated and/or analyzed during the current study are available from the corresponding author upon reasonable request.

## Author contributions

FG and CY have made a substantial contribution to the concept and design of the article. JH, YS and XC analyzed data for the article and drafted the article. HH, KQ and JL revised it critically for important intellectual content. All authors approved the version to be published, and they have agreed to be accountable for all aspects of the work in ensuring that questions related to the accuracy or integrity of any part of the work are appropriately investigated and resolved.
