# Supplementary figures and images for: A novel pyroptosis gene expression-based risk score for survival in gastric cancer
Source: Front Endocrinol (Lausanne). 2023 Jan 30;14:1120216. doi: 10.3389/fendo.2023.1120216 (PMC9922719; doi:10.3389/fendo.2023.1120216)

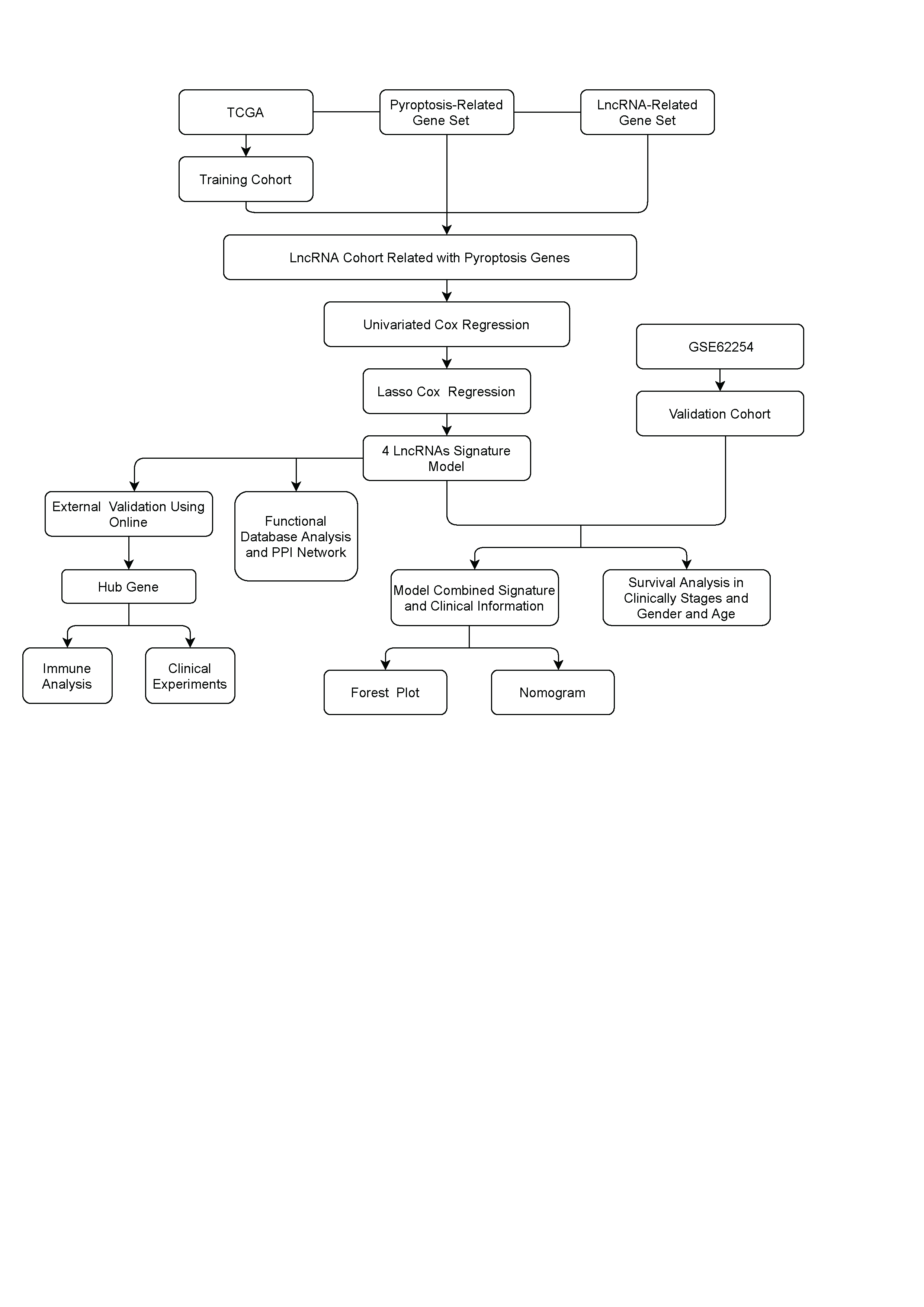

Supplement: Supplementary file 1 [file Image_1.tif]

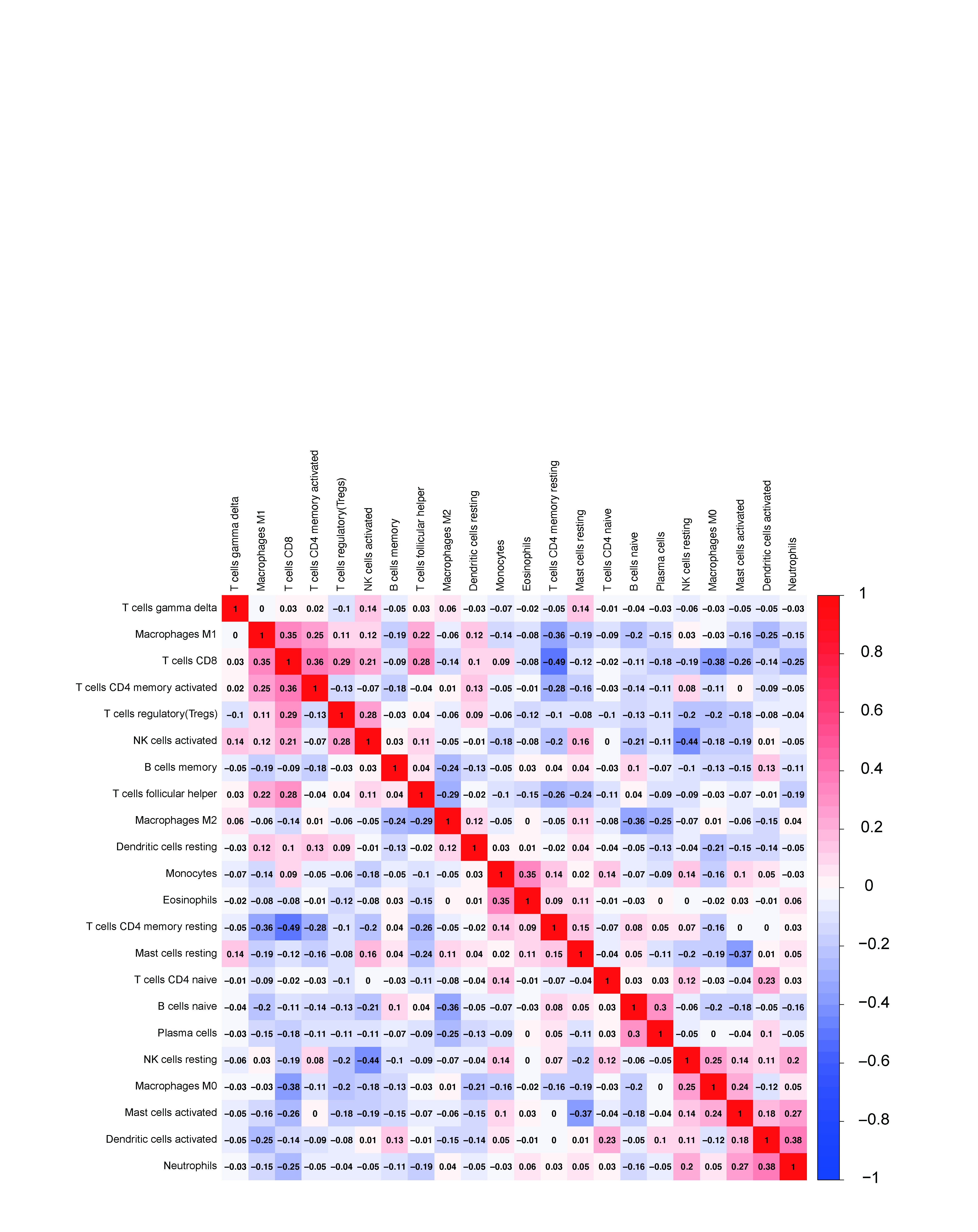

Supplement: Supplementary file 2 [file Image_2.tif]
